# Plasmon-Enhanced Fluorescence Determination of Tamsulosin Using Silver Nanoparticles: A Green Analytical Strategy

**DOI:** 10.1007/s10895-025-04545-y

**Published:** 2025-09-17

**Authors:** Mariam Rashad, Fawzi Elsebaei, Zeinab Awad Sheribah, Mohamed Ibrahim Walash

**Affiliations:** Faculty of Pharmacy, Pharmaceutical Analytical Chemistry Department, Mansoura University, Mansoura, 35516 Egypt

**Keywords:** Tamsulosin, Fluorescence, Spectofluorimetry, Silver nanoparticles, Greenness assessment, Whiteness assessment.

## Abstract

A novel, simple, and highly sensitive spectrofluorometric method was developed for the quantification of Tamsulosin (TAM) in methanol, utilizing silver nanoparticles (AgNPs) as fluorescence enhancers. The AgNPs formed colloidal dispersions with particle sizes ranging between 5 and 50 nm, enhancing TAM’s native fluorescence through a catalytic interaction. Fluorescence emission was measured at 315 nm after excitation at 285 nm, and the method exhibited linearity in the range of 100–500 ng/ml. The method was successfully applied to the assay of two commercial TAM capsule formulations obtained from a local pharmacy. Validation was carried out in accordance with International Council for Harmonisation (ICH) guidelines, confirming the method’s accuracy, precision, and robustness. Furthermore, the ecological impact of the proposed method was evaluated using greenness assessment tools, including AGREE and the Analytical Eco-Scale, demonstrating its sustainability in terms of solvent use, chemical consumption, energy efficiency, and waste reduction.

## Introduction

**Fig. 1:**
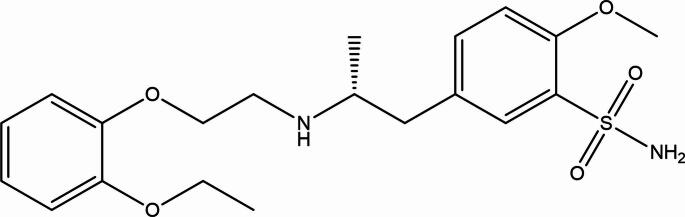
Tamsulosin chemical structure

Tamsulosin (TAM), with the chemical formula C₂₀H₂₈N₂O₅S and the IUPAC name (R)−5-(2-{[2-(2-ethoxyphenoxy)ethyl]amino}propyl)−2-methoxybenzene-1-sulfonamide (Fig. 1), is a selective α₁-adrenergic receptor blocker. It acts on the smooth muscle of the prostate and bladder neck, reducing urethral resistance and improving urinary flow. Tamsulosin is widely prescribed for the management of benign prostatic hyperplasia (BPH) symptoms in adult males [1–3].

TAM is an official drug listed in the British Pharmacopoeia (BP), European Pharmacopoeia (Ph. Eur.), and the United States Pharmacopoeia (USP) [4–6] presented under different trade names like Flomax^®^ and tamsulosin^®^ 0.4 mg oral capsules. There are several analytical approaches used to determine and quantify TAM presented in reported articles.

These analytical approaches include different spectrophotometric methods [7–15], fluorometric methods [16–20], chromatographic methods [21–29], capillary electrophoresis [30, 31]and electroanalytical methods [32–34]. TAM is analyzed either alone or with other common co-administrated drugs such as dutasteride, finasteride, tadalafil and tolterodine. The above-mentioned methods have different drawbacks that vary between being time and material consuming, complicated and require highly skilled analysts.

In recent years, nanoscience has become a prominent area of research in the field of analytical chemistry. The unique physicochemical properties of noble-metal nanoparticles, such as gold and silver, have enabled their widespread use in diverse fields, including drug delivery, biosensing, food packaging, and environmental monitoring.

In analytical applications, silver nanoparticles (AgNPs) have gained particular attention due to their surface plasmon resonance (SPR) and catalytic behavior, which can enhance signal responses in spectroscopic methods, including fluorescence. Their small size, high surface-to-volume ratio, and tunable optical properties make them ideal candidates for sensitive detection platforms.

In this study, we focus on the use of AgNPs in pharmaceutical analysis, aiming to improve the fluorimetric quantification of Tamsulosin (TAM) through nanoparticle-induced enhancement of its native fluorescence [35, 36].

Localized surface plasmon resonance (LSPR) arises from the collective oscillation of conduction electrons on the surface of metallic nanoparticles when excited by light at specific wavelengths. This phenomenon is responsible for the distinct optical properties of nanoparticles, including their size-dependent color and absorption behavior, and is influenced by particle size, shape, and surrounding refractive index.

Among noble metals, silver nanoparticles (AgNPs) are particularly favored due to their strong LSPR response, chemical stability, cost-effectiveness, and excellent catalytic and optical properties. These characteristics enable their use in diverse analytical techniques, including surface-enhanced Raman scattering, electrochemical sensing, and fluorescence modulation.

In the present study, AgNPs were utilized to enhance the native fluorescence of Tamsulosin (TAM), enabling a novel, simplified, and highly sensitive spectrofluorometric method. This approach not only improves analytical performance but also aligns with green chemistry principles, making it a valuable contribution to pharmaceutical analysis.

## Experimental

### Equipment

**Fig. 2:**
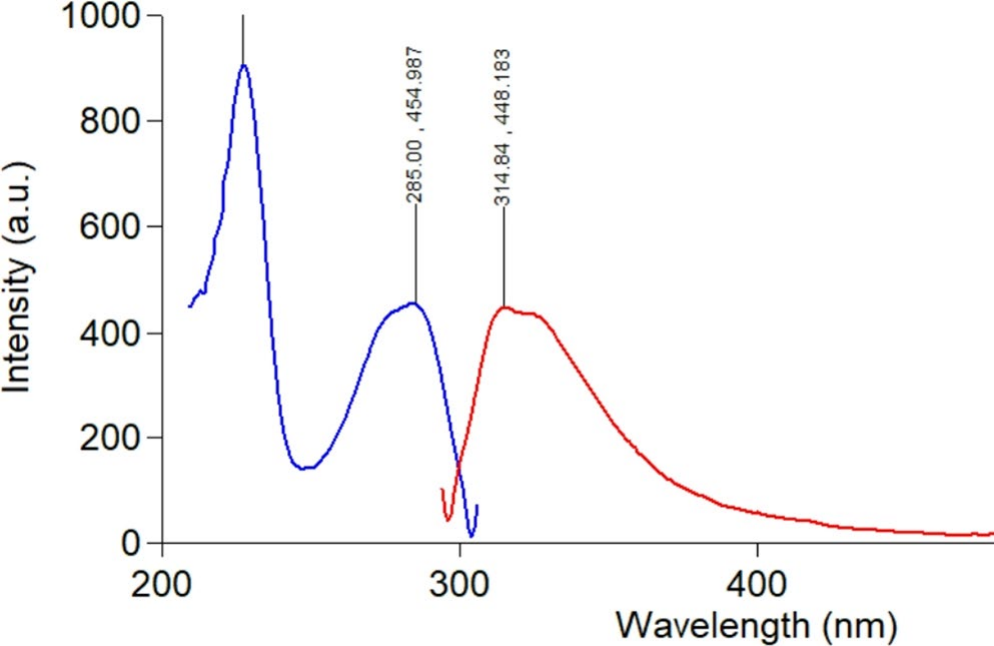
Excitation and emission spectrum of 500 ng/ml TAM in methanol where blue is the excitation spectrum at 285 nm and red is the emission spectra at 315 nm

- Using a Xenon flash lamp, an Agilent Technologies Cary Eclipse fluorescence spectrophotometer was used. An 800-volt high voltage mode was used, and the slit width was 5 nm. Excitation/emission wavelengths of 285/315 nm were employed (Fig. 2). There was a 19% smoothing factor.

- A double-beam 1601 Shimadzu UV-Visible Spectrophotometer was used to make the spectrophotometric observations.
- Electron microscope for transmission (TEM), JEOL, JSM-2100 (Tokyo, Japan). On a 200-mesh carbon-coated Cu grid, the sample was loaded, and 200 kV was used for the examination.
-pH meter: Consort, Belgium, P-901.
- Magnetic stirrer: a product of South Korea’s Daihan Scientific Co., Ltd.
- Thermostatically controlled water bath.

### Materials and Reagents

Authentic samples of Tamsulosin hydrochloride with 99.99% purity was obtained from National Organization for Drug Control and Research (NODCAR) in Egypt.

Fisher Scientific, Bishop Meadow Road, Loughborough, LE11 5RG, UK, provided the acetonitrile, ethanol, and methanol (HPLC grade).

We bought silver nitrate (AgNO3), and sodium borohydride (NaBH₄) from Sigma-Aldrich (USA).

The following surfactants were purchased from El Nasr Chemical Co. in Egypt: cetrimide, sodium dodecyl sulfate (SDS), carboxymethyl cellulose (CMC), and Tween 80. All were made as 0.5% w/v in water to examine their effect on the fluorescence intensity of the TAM and AgNPs.

The buffers were made using boric acid, sodium hydroxide, sodium acetate, and acetic acid, all of which were purchased from El-Nasr Pharmaceutical Chemicals Company (ADWIC), Egypt.

Tamsul and tamsulin Capsules labeled to contain 0.4 mg of TAM per capsule were purchased from a local pharmacy.

A stock solution (100 µg/ml) of TAM was prepared by dissolving 10 mg of TAM in 100 ml of methanol in a volumetric flask. We got different concentrations of working solutions by diluting stock solutions to required concentrations using methanol.

### Silver Nanoparticles Preparation

The preparation depends on chemical reduction of silver nitrate using sodium borohydride without the help of organic stabilizers. The synthesis was completed in a pre-washed flask with concentrated nitric acid. By thoroughly washing the flasks with distilled water, the residual acid on the glass walls was eliminated.

An ice-cold solution of 1 mmol/L of sodium borohydride (NaBH₄) was prepared and placed under vigorous magnetic stirring. To this, 1 mmol/L AgNO₃ was added in a 6:1 (NaBH₄:AgNO₃, v/v) ratio. Upon mixing, a pale-yellow color appeared within the first 20 s, gradually developing into a brownish-yellow colloid, indicating the formation of AgNPs.

To prevent photodegradation, the resulting colloidal solution was stored in a dark, refrigerated environment until further use [37].

### Buffer Preparation

By mixing 0.2 M sodium acetate with 0.2 M acetic acid and adjusting the mixture to the appropriate pH using a pH meter, a 0.2 M acetate buffer with a pH range of 3-5.5 was prepared.

By mixing 0.2 M sodium hydroxide and 0.2 M boric acid and adjusting the mixture to the desired pH using a pH meter, 0.2 M borate buffer with a pH range of 6–10 was prepared.

### General FL Procedure

The optimum FL conditions were studied at room temperature for the FLs used to determine TAM in the presence of AgNPs. Working solution aliquots were transferred into 10 ml volumetric flasks as shown in Table 1. In the presence of 2.8 × 10^−6^ M AgNPs, the fluorescence detection was carried out at room temperature at λem 315 nm after λex of 285 nm. The graph for calibration was plotted, and by using the regression equation, TAM concentration was evaluated.

**Table 1:** Analytical performance data for the proposed method

Validation parameter	TAM only	TAM with AgNPs
λ_ex_ - λ_em_	285–315 nm	
Solvent	Methanol	
Linearity range (ng/mL)	100–500 ng/mL	
Intercept (a)	−9.4	3.5
Slope (b)	0.81	1.069
Correlation coefficient (r)	0.9998	0.9991
S.D. of residuals (S_y/x_)	2.898	8.26
S.D. of intercept (S_a_)	3.04	8.66
S.D. of slope (S_b_)	0.009	0.026
% RSD	1.829	1.961
% Error	0.821	0.878
Limit of detection, LOD (ng/mL)	12.38	26.74
Limit of quantitation, LOQ (ng/mL)	37.53	81.04

### Pharmaceutical Dosage Form Analysis

A total of 25 capsules containing 10 mg TAM (each containing 0.4 mg) of precisely weighed ground powder were added to a 25 mL volumetric flask; then 20 mL of methanol was added, given 45 min of sonication, and completed to the mark with the same solvent. The combination was filtered to create a 400 µg/ml stock solution. Additional dilution using the same solvent was carried out to obtain working standard solutions to be examined while adhering to the general procedures described under establishing the calibration curve. We used the corresponding regression equation to determine the contents of each dose form.

## Results and Discussion

### AgNPs Characterization

**Fig. 3:**
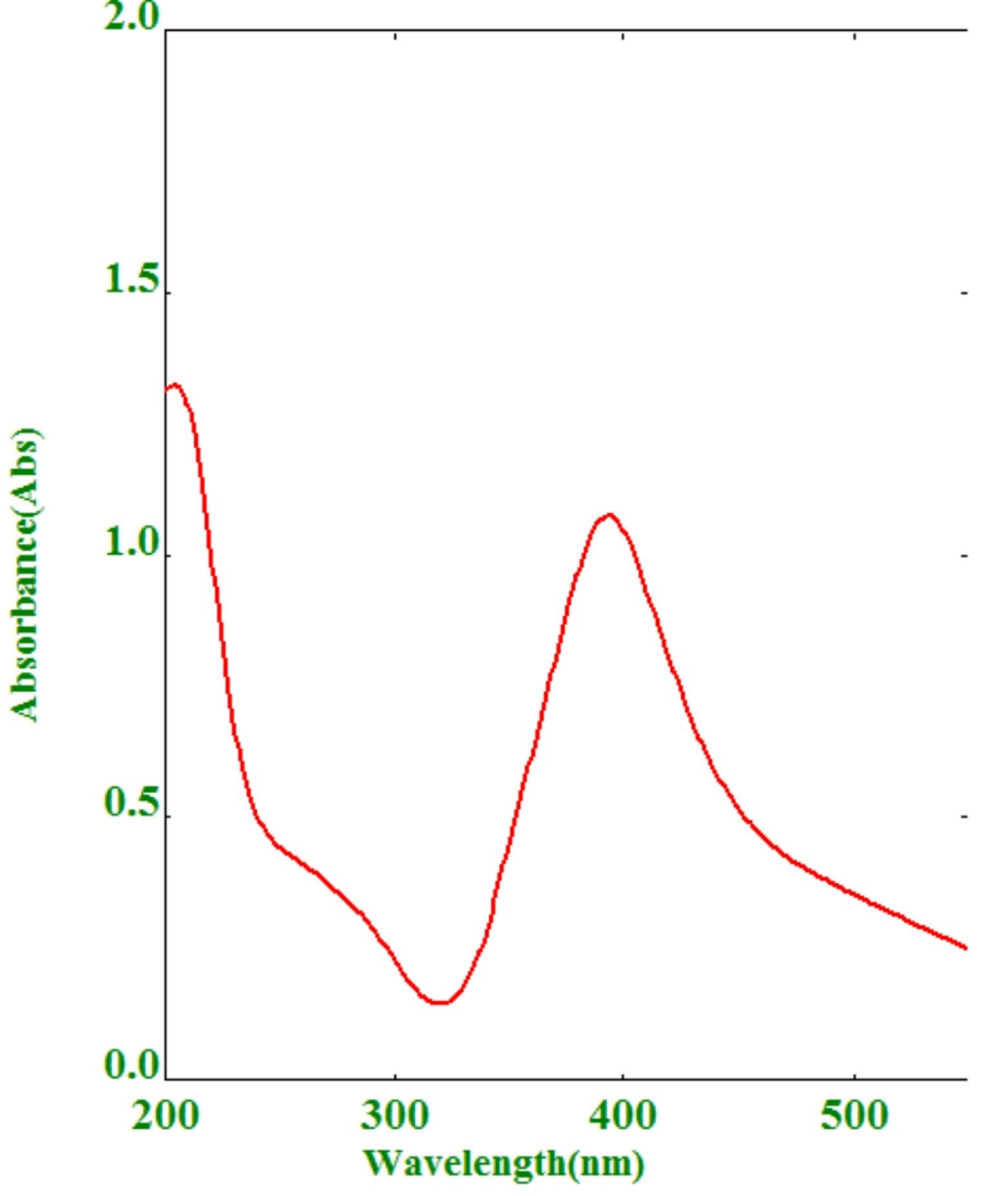
UV spectrum of 1.4 × 10^−4^ M AgNPs showing maximum absorbance at 400 nm

Figure 3 shows the absorption spectrum of AgNPs at 400 nm of 1.4 × 10^−4^ M concentration.

**Fig. 4:**
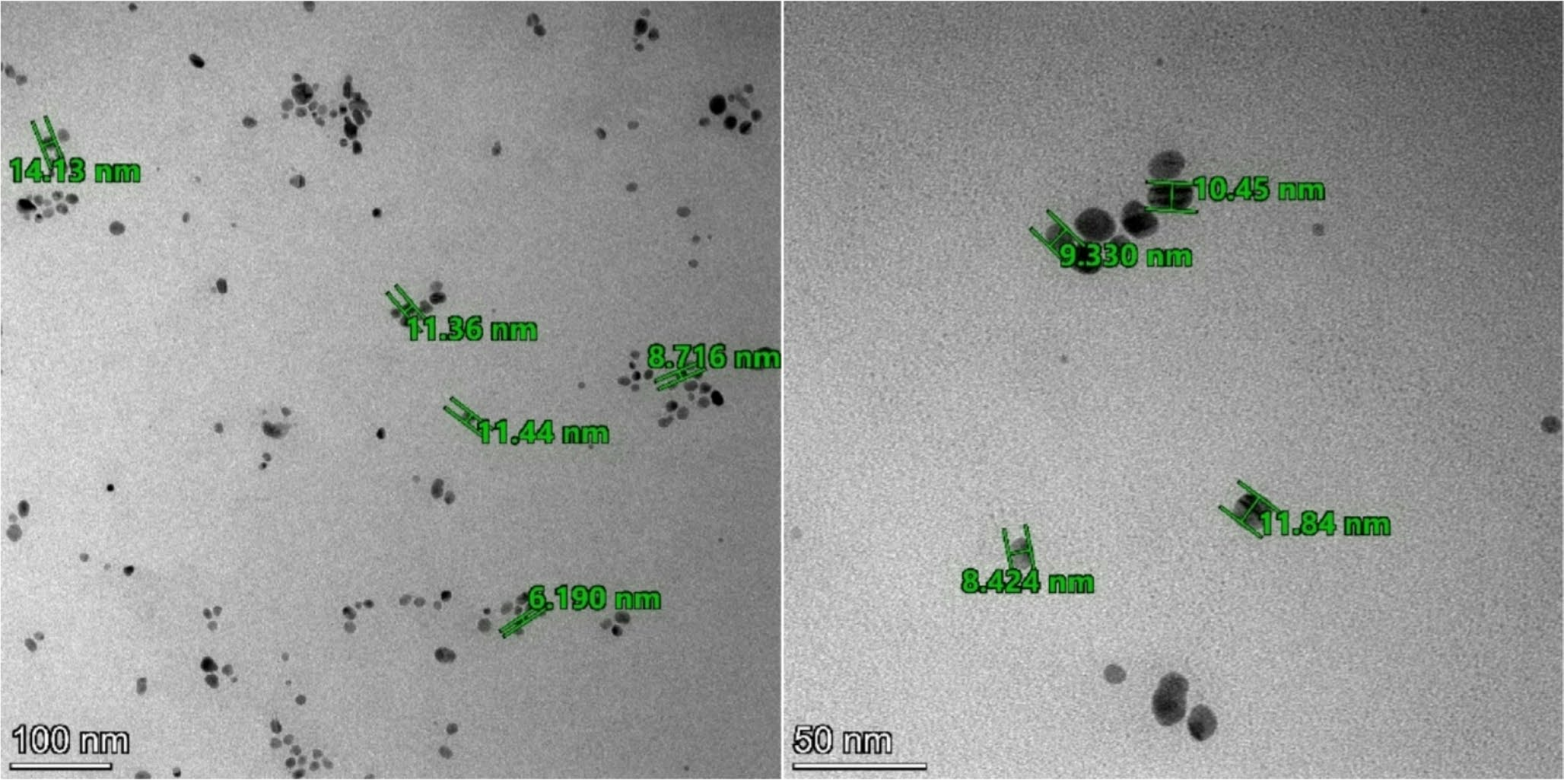
TEM images of AgNPs

Size, shape, and concentration of AgNPs affect their optical characteristics. The microscopic evaluation of AgNPs was obtained using TEM (Fig. 4). Which shows spherical-shaped nanoparticles.

### Optimization of Experimental Conditions

#### Effect of AgNPs Concentration and Volume

**Fig. 5:**
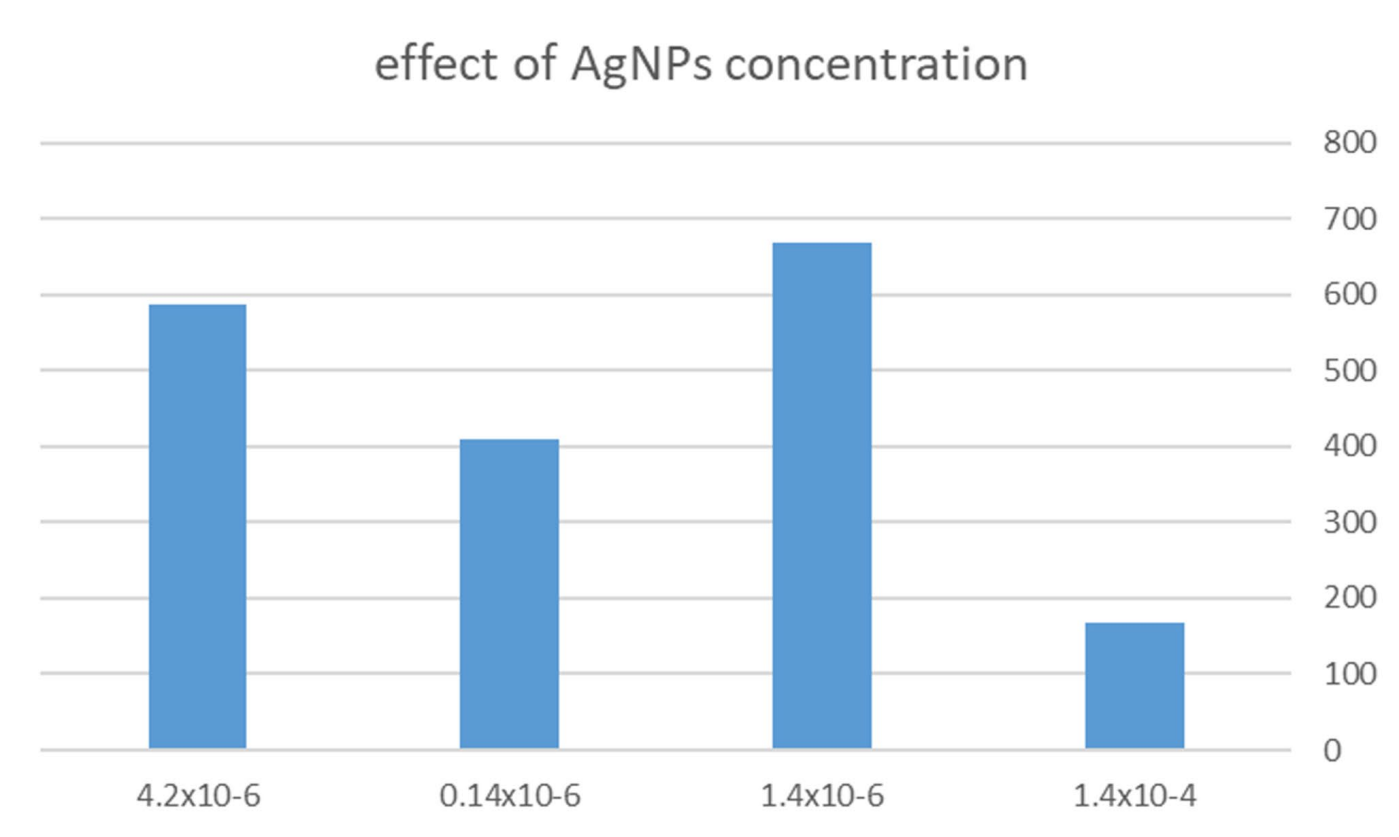
Effect of AgNPs concentration on fluorescence intensity of 500 ng/ml TAM. (note that all numbers in the figure below are multiplied by 10^−6^)

AgNPs concentration effect was studied within the range of (1.4 × 10^−8^ – 1.4 × 10^−6^) M on 500 ng/ml TAM Fig. 5. and volume range 1–5 ml. Highest Fluorescence intensity was achieved using 2 ml volume of 1.4 × 10^−6^ M concentration.

**Fig. 6:**
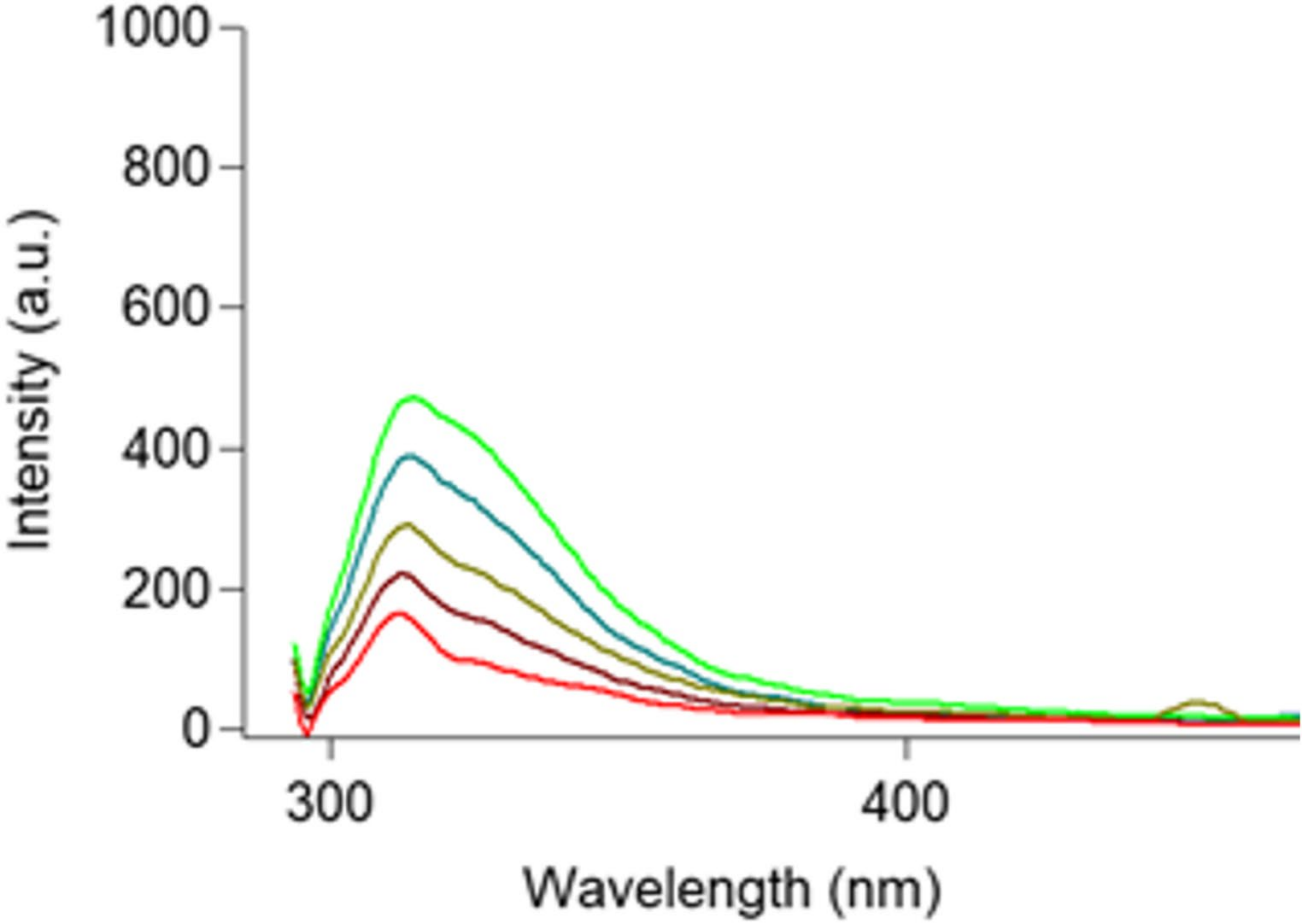
Emission spectrum of increasing concentration of TAM where light green resamples 500 ng/ml, dark green resamples 400 ng/ml, olive green resamples 300 ng/ml, dark red resamples 200 ng/ml and red resamples 100 ng/ml (from bottom to top 100,200,300,400 and 500ng/ml)

**Fig. 7:**
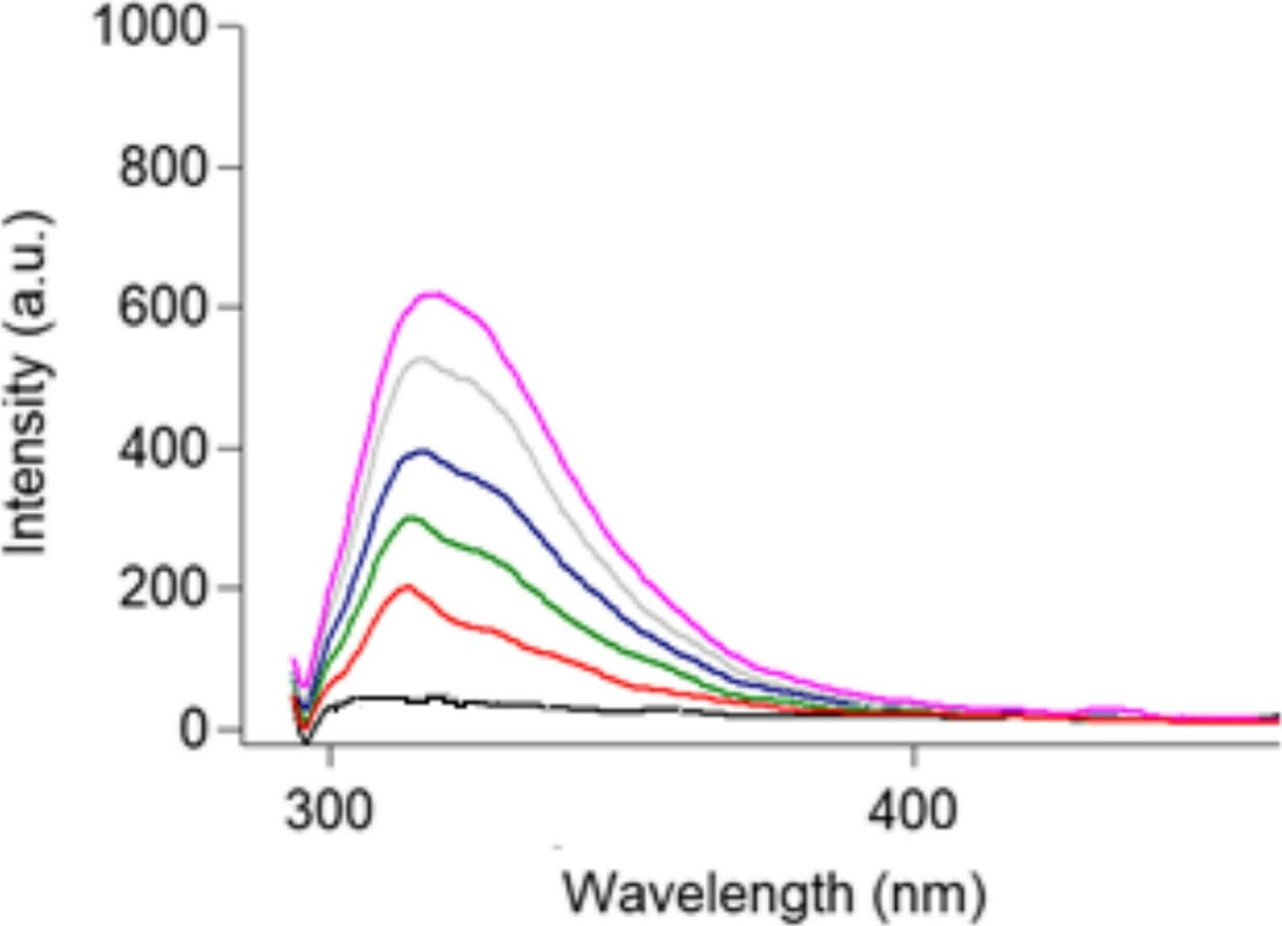
Emission spectra of increasing concentration of TAM with 2.8 × 10^−6^ M where black resamples blank, red resamples 100 ng/ml, green resamples 200 ng/ml, blue resamples 300 ng/ml, grey resamples 400 ng/ml and pink resamples 500 ng/ml (from bottom to top blank,100,200,300,400 and 500 ng/ml)

Figure 6 shows the fluorescence spectra of TAM dissolved in methanol only within (100–500) ng/mL range. While Fig. 7 shows the effect of 2.8 × 10^−6^ M AgNPs on the same range of TAM concentrations, confirming the significant enhancement in fluorescence intensity across the same concentration range.

#### Effect of Diluting Solvents

**Fig. 8:**
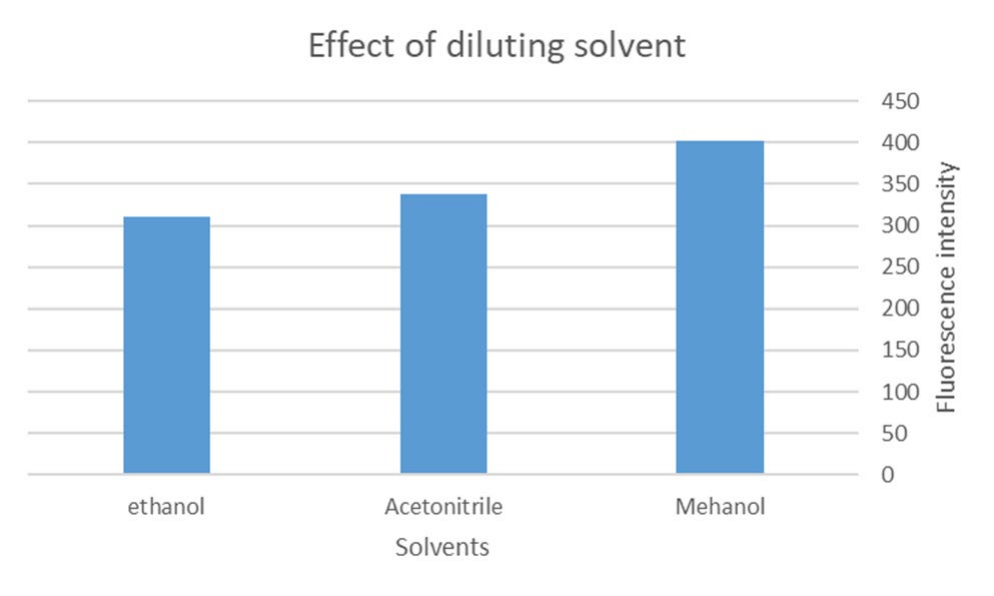
Effect of diluting solvents on Fluorescence intensity of 450 ng/ml TAM mixed with 2.8 × 10^−6^ M AgNPs

The effect of solvents on 450 ng/ml TAM mixed with 2.8 × 10^−6^ M AgNPs fluorescence intensity was studied using different solvents such as methanol, acetonitrile, and ethanol. Figure 8. Water as a solvent was omitted as TAM is sparingly soluble in water at 0.00655 mg/ml.

Methanol was found to be the optimum diluting solvent.

#### Effect of pH

The acid–base behavior of Tamsulosin (TAM) was considered during optimization, with pKa values of 9.93 (strongest acidic) and 9.28 (strongest basic). The effect of pH on TAM fluorescence intensity was studied using two buffer systems:

- Acetate buffer (pH 3.0–5.5)
- Borate buffer (pH 6.0–10.0)

**Fig. 9:**
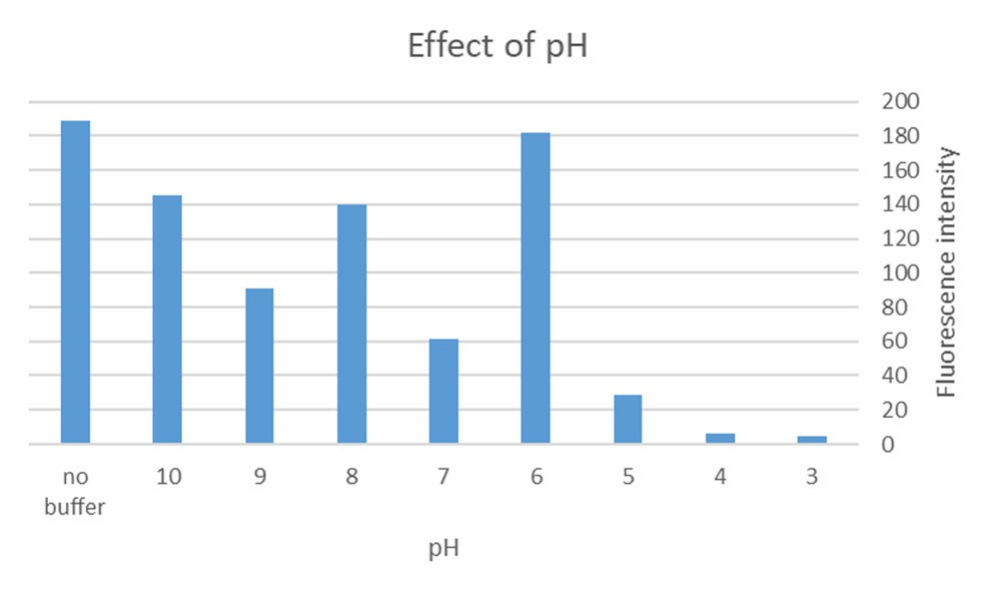
Effect of pH on 150 ng/ml TAM mixed with 2.8 × 10^−6^ M using 1 ml of prepared buffer concentration

Experiments were conducted using 150 ng/mL TAM mixed with 2.8 × 10⁻⁶ M AgNPs, diluted in methanol. As shown in Fig. 9, no significant enhancement in fluorescence intensity was observed across the tested pH ranges when buffers were used.

Notably, the absence of any buffer led to the highest and most stable fluorescence signal, likely due to the limited solubility of TAM in aqueous buffer media and potential quenching effects associated with buffer ions. Therefore, unbuffered methanol was selected as the optimal solvent system for subsequent analyses.

#### Effect of Surfactant

**Fig. 10:**
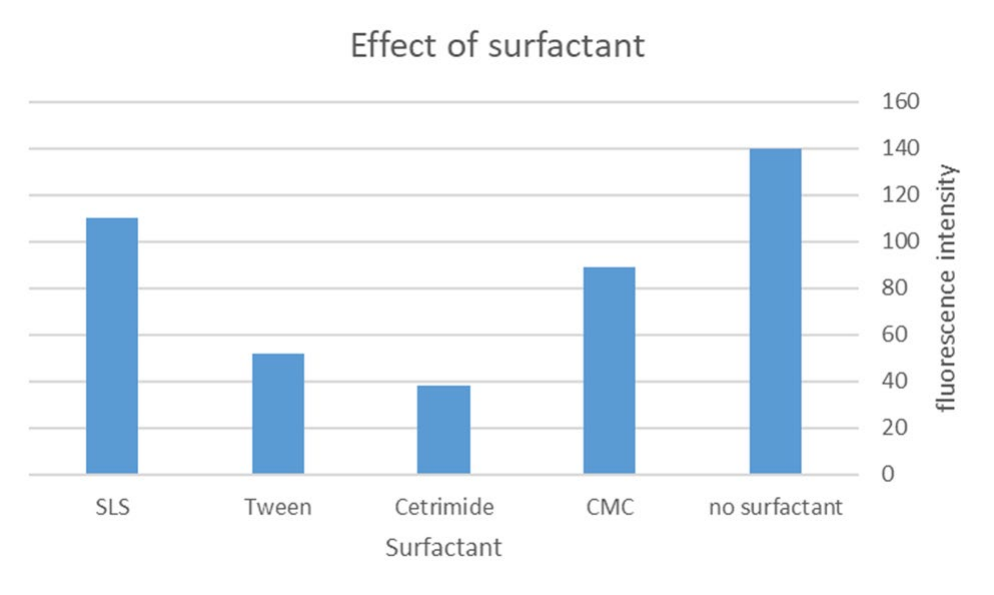
Effect of different surfactants on 100 ng/ml TAM mixed with 2.8 × 10^−6^ M using 1 ml of each prepared surfactant concentration

To evaluate the influence of micellar environments on TAM fluorescence, various surfactants were investigated at concentrations above their critical micelle concentration (CMC). The study was performed using 100 ng/mL TAM mixed with 2.8 × 10⁻⁶ M AgNPs, diluted in methanol. The results are illustrated in Fig. 10.

Different types of surfactants were tested, including anionic, cationic, and nonionic agents (e.g., Sodium dodecyl sulfate (SDS), cetyltrimethylammonium bromide (CTAB), and Tween-80, respectively). However, the addition of surfactants led to either no enhancement or a decrease in fluorescence intensity, likely due to competitive binding, micelle encapsulation, or quenching interactions with the fluorophore or nanoparticles.

Interestingly, the highest and most consistent fluorescence response was obtained in the absence of any surfactant, supporting the use of pure methanolic medium without additional micellar modifiers in the final protocol.

#### Effect of Reaction Time

The effect of reaction time between AgNPs and TAM on fluorescence intensity was studied. It reached a constant value within seconds and remained constant for more than half an hour.

#### Effect of Temperature

The effect of temperature on fluorescence intensity was investigated. Using a temperature range of 25–100 °C with the help of a thermostatically controlled water bath. The increase in temperature leads to a decrease in fluorescence intensity, as higher temperatures promote enhanced internal conversion and the nonradioactive deactivation of the excited singlet state [38], thus all tests were investigated at room temperature.

### Validation of the Proposed Method

Validation was done under ICH recommendations [39].

#### Linearity, Limit of Detection (LOD) and Limit of quantification (LOQ)

Plotting FI against concentrations of TAM allowed the construction of the TAM calibration graphs. The intensity of TAM fluorescence increased linearly upon the addition of AgNPs by increasing drug concentration. The results showed a rectilinear range (100–500 ng/ml) with an excellent correlation coefficient of 0.9991. The minimal level at which the analyte can be reliably identified was established to estimate the LOD, which equals 26.74 ng/ml. While LOQ was determined under specified experimental conditions, the minimum quantity of analyte in a sample that can be determined with reasonable precision and accuracy was found to be 81.04 ng/ml.

LOD and LOQ were calculated according to ICH guidelines [39] using the equations:

$$LOD=3.5\times Sa/b\;and\;LOQ=10\times Sa/b$$

where Sa is standard derivatization of the intercept and b is the slope of the calibration curve. Table 1 summarized these data.

#### Accuracy

To evaluate the accuracy and applicability of the proposed spectrofluorimetric method, the results obtained for TAM determination in its pure form were statistically compared with those from a previously reported method [28]. The comparison was carried out using Student’s t-test and variance ratio F-test, and the results are presented in Table 2 (note that each concentration was analyzed three times, and the values presented in the table represented the mean of the three dimensions). The calculated values of t and F did not exceed the theoretical values at the 95% confidence level, indicating no significant difference between the proposed and reported methods in terms of accuracy and precision. This confirms that the proposed method is reliable and suitable for routine quality control analysis [40].

**Table 2:** Application of the proposed method to the determination of TAM in its Raw material

	Proposed method			Reported method [28]		
	Amount taken (ng/mL)	Amount found (ng/mL)	Percentage found	Amount taken (µg/mL)	Amount found (µg/mL)	Percentage found
TAM	100	101.45	101.45	200	198.78	99.39
	200	199.72	99.86	300	309	103
	300	292.41	97.47	400	393.84	98.46
	400	410.11	102.53	500	497.15	99.43
	500	496.28	99.26	600	606.60	101.1
Mean ± S.D.			100.114 ± 1.963			100.276 ± 1.796
t^b^	0.14(2.31)					
F^b^	1.19(6.39)					

Each value represents the mean of three replicate measurements

#### Robustness

Through minor adjustments to the method parameters that are discussed above, the robustness of the FL detection techniques for TAM estimation was examined. These adjustments did not impact the FIs of the proposed systems. 

#### Precision

Both inter-day and intra-day tests were examined. Three distinct TAM concentrations were examined three times (*n* = 3), and the collective findings were reported as % RSD in Table 3.

**Table 3:** Inter-day and intra-day precision data for TAM using the proposed method *(Methanol as solvent)

Concentration of TAM (ng/mL)	Intra-day precision			Inter-day precision		
	Mean ± SD	RSD (%)	Standard error	Mean ± SD	RSD (%)	Standard error
100.0	99.97 ± 1.13	1.13	0.8	100.13 ± 1.80	1.80	1.04
300.0	100.07 ± 0.91	0.906	0.523	100.23 ± 1.17	1.17	0.673
500.0	99.94 ± 0.57	0.567	0.327	100.57 ± 1.91	1.90	1.09

### Analytical Application

This study was successfully applied to TAM hard capsules (tamsul 0.4 mg) and (tamsulin 0.4 mg) obtained from a local pharmacy (Table 4).

**Table 4:** Analysis of capsule containing TAM and AgNPs using the proposed method

Tablets name	Developed method		Reported method [28]		
	Concentration (ng/mL)	% Found ^a^	Concentration (µg/mL)	% Found ^a^	
Tamsul cap	100	101.79	100	98.37	
	400	98.87	400	99.28	
	500	100.37	500	100.52	
Mean		100.34		99.39	
± S.D.		1.46		1.079	
*t* ^b^	0.91 (2.78)				
*F* ^b^	1.83 (19.00)				
Tamsulin cap	100	99.02	100		101.15
	400	101.01	400		98.94
	500	100.20	500		99.08
Mean		100.08			99.72
± S.D.		1			1.24
*t* ^b^	0.38 (2.78)				
*F* ^b^	0.65 (0.05)				

## Environmental Assessment

### Greenness Assessment

The goal of green analytical chemistry is to make analytical processes safer for both people and the environment. Numerous factors are considered when evaluating the greenness of an analytical method, including the amount and toxicity of the chemicals used, waste produced, energy needs, the number of steps in the process, automation, and downsizing. We used the AGREE technique, which converts the 12 important principles of green analytical chemistry into a single 0–1 scale for evaluation criteria. The ultimate score is determined based on the important principles. The end product is a pictogram that displays the user-assigned weights, the final score, and the success of the analytical process in each criterion [41]. With an Analytical Eco-scale score of 84 and an AGREE score of 0.62, as shown in Table 5, this novel approach showed a high level of greenness.

**Table 5:** Greenness assessment

Tools of assessment	TAM With AgNPs effect investigation:	
Analytical Eco Scale	84	
	Reagents:	Penalty points
	methanol	4
	AgNo_3_	4
	NaBH_4_	8
	Energy for fluorescence:	0
	waste	0
	Occupational hazards:	0
	Total penalty points	16
AGREE		

### Whiteness Profile

To further demonstrate the sustainability and impact of the proposed spectrofluorometric method, a whiteness assessment was performed based on the principles of White Analytical Chemistry (WAC) [42, 43]. Unlike traditional green assessments, the WAC approach provides a more holistic evaluation by integrating analytical efficiency, environmental impact, and practical applicability.

The method shows excellent analytical performance with high sensitivity (LOD: 26.74 ng/mL with AgNPs), linearity (*r* = 0.9991), and precision (%RSD = 1.961). It also involves minimal sample preparation with short analysis time (< 1 min/sample), low waste generation (5–9 mL methanol per sample), and avoids hazardous derivatization or pretreatment steps. The use of AgNPs offers sensitivity enhancement without the need for complex instrumentation with average whiteness score of 89 as shown in Table 6.

**Table 6:** Whiteness profile:

category	principle	assessment	score
green	Low reagent consumption	5–9 ml mw = ethanol per sample and minimal AgNPs used	90
	Waste generation	No hazardous. Methanol is disposed in lab sink	85
	Reagent toxicity	No oxidizers or toxic solvent. Only methanol which is of moderate risk	70
analytical	Sensitivity and precision	LOD = 26.74 ng/ml %RSD = 1.96% *r* = 0.9991	95
	accuracy	T and f test confirm no significant bias	90
	Selectivity	Showed no interference from formulation matrix	90
Practical	Analysis time	< 1 min per sample	100
	Throughput and ease	Simple mixing and no derivatization	95
	Instrument and energy	Xenon lamp fluorimeter	85
	Operator safety	Standard lab and no fume hood needed.	90
Average whiteness score			89

The detailed whiteness profile is presented in Table X, showing high scores in most criteria, especially in environmental and practical aspects, confirming that the method is not only green but also “white” — modern, sustainable, and fit for real-world application.

## Method Novelty and Comparative Assessment

Table 7 shows the comparison of this method with previously published method [18].

**Table 7:** Comparison with previously published method:

Parameter	Proposed method	Previously published method[18]
Linearity range (ng/ml)	100–500	750–3500
LOD (ng/ml)	12.38 (native) 26.74 (with AgNPs)	170 − 130
Derivatization	No	required
Nanomaterials used (e.g., AgNPs)	AgNPs	No
Environmental assessment	Green and white metrics were used	No

The previously published method was based on derivative and ratio techniques and validated for TAM in binary dosage forms (range 0.75–3.50 µg/mL). Compared to this method, the proposed AgNPs‑enhanced method achieves higher sensitivity (LOD 26.74 ng/mL) within a broader linear range (100–500 ng/mL), without the need for derivatization or complex instrumentation. Moreover, our method includes green and whiteness assessments (AGREE, Eco‑Scale, WAC), which were not addressed by El‑Kimary et al., thereby confirming the novelty practicality, and eco-friendly profile of the proposed approach.

## Conclusion

A green, simple, and highly sensitive spectrofluorometric method was successfully developed and validated for the determination of Tamsulosin (TAM) in both bulk powder and commercial capsule formulations. The method relies on the fluorescence enhancement induced by silver nanoparticles (AgNPs), whose plasmonic and catalytic properties significantly amplified TAM’s native emission signal.

The proposed method demonstrated excellent linearity over the range of 100–500 ng/mL, along with high precision and repeatability. Comparative statistical analysis with a reported method confirmed its accuracy and analytical reliability.

In addition to its analytical performance, the method aligns with green chemistry principles, as confirmed by AGREE and Analytical Eco-Scale assessments. Its simplicity, cost-effectiveness, and environmental safety make it highly suitable for routine quality control of TAM in pharmaceutical settings.

The proposed method not only demonstrates high sensitivity and greenness but also shows superior performance compared to previously published fluorimetric methods for Tamsulosin, confirming its novelty and practical applicability.

## Data Availability

No datasets were generated or analysed during the current study.
